# A subnational profiling analysis reveals regional differences as the main predictor of ITN ownership and use in Nigeria

**DOI:** 10.1186/s12936-019-2816-9

**Published:** 2019-05-28

**Authors:** Andrew Andrada, Samantha Herrera, Uwem Inyang, Audu Bala Mohammed, Perpetua Uhomoibhi, Yazoumé Yé

**Affiliations:** 1 MEASURE Evaluation, ICF, 530 Gaither Road, Suite 500, Rockville, MD 20850 USA; 2 Save the Children, 899 North Capitol Street, NE, #900, Washington, DC 20002 USA; 3 President’s Malaria Initiative/United States Agency for International Development, Plot 1075 Diplomatic Drive, Central District Area, Abuja, Nigeria; 4 National Malaria Elimination Programme, Federal Ministry of Health, 1st Floor, Abia Plaza, 1 Avenue, Cadastral Zone A0, Central Business District, Abuja, Nigeria

**Keywords:** Insecticide-treated nets, Net use, Malaria prevention and treatment, Sub-Saharan Africa, Nigeria, Chi square automatic interaction detection

## Abstract

**Background:**

To reduce the malaria burden in Nigeria, the country is scaling up prevention and treatment interventions, especially household ownership and use of insecticide-treated nets (ITNs). Nevertheless, large gaps remain to achieve the goals of the National Malaria Strategic Plan 2014–2020 of universal access to ITNs and their increased use. To inform the targeting of intervention strategies and to maximize impact, the authors conducted a sub-national profiling of household ITN ownership and use in the general population to identify key predictors of ITN ownership and use, and the sub-groups that are at higher risk of low ITN coverage and use.

**Methods:**

The authors conducted a secondary analysis of data from the 2015 Nigeria Malaria Indicator Survey. Using the Chi square automatic interaction detector (CHAID) and multiple logistic regression analysis, the authors examined the key predictors of ITN ownership and use in the general population throughout Nigeria.

**Results:**

The CHAID models identified region of the country as the best predictor of household ownership of at least one ITN and its use in the general population, with higher ownership and use observed in the northern regions. The odds of a household owning an ITN were five times greater in the North West region compared with the North Central region (odds ratio [OR] = 5.47, 95% confidence interval [CI] 4.46–6.72, p < 0.001). The odds of ITN use were two times greater for those living in the North West region compared with the North Central region (OR = 2.04, 95% CI 1.73–2.41, p < 0.001). Other significant predictors were household size, head of household education level, household wealth quintile, and place of residence. The CHAID gain index results identified households in the South West, North Central and South Central regions with low ITN ownership, and the general population in the South South, South East and North Central regions with low ITN use.

**Conclusions:**

This study reveals regional differences in ITN ownership and use in Nigeria. Therefore, the findings from this analysis provide evidence that could inform the NMEP to better target future campaign and routine distribution of ITNs, to achieve universal access and increased use by 2020 in Nigeria.

## Background

Malaria is a major public health issue in Nigeria, with 100% of the population at risk [1]. The country has the highest proportion of malaria cases (27%) and estimated malaria deaths (30%) globally; nationally, the malaria burden accounts for 60% of outpatient visits to health facilities and 30% of child mortality [2]. The geographic spread of the malaria burden is heterogeneous in the country, with the highest prevalence among children ages 6 to 59 months in the North Central, North East and North West regions, and the lowest prevalence in the South East region [3].

To reduce the malaria burden, the Government of Nigeria, through the National Malaria Elimination Programme (NMEP) and in collaboration with partners, is scaling up malaria prevention and treatment interventions in line with the goals of the National Malaria Strategic Plan (NMSP) 2014–2020. Under this strategic plan, the NMEP is striving to increase insecticide-treated net (ITN) ownership coverage and raise awareness to increase ITN use [4]. The NMEP employs a mixed-model approach for ITN distribution that includes free mass distribution campaigns and continuous distribution of ITNs to supplement the mass campaigns. Continuous distribution relies on several routine health service delivery channels: immunization campaigns; antenatal care (ANC); the integrated maternal, newborn, and child health week; school-based distribution; community-based distribution; and, distribution through the commercial sector [3]. Between 2014 and 2016, more than 60 million ITNs were distributed, which helped increase household ownership of at least one ITN from 8% in 2008 to 69% in 2015 [2, 3, 5]. The majority of households (77%) received their ITNs through free mass distribution campaigns [3]. Following the recommendation by the WHO, the ITNs distributed by the NMEP have been long-lasting, insecticide-treated nets (LLINs) [6]. LLIN brands distributed in these mass campaigns include Duranet, Iconlife, Interceptor, Netprotect, Olyset and Permanet [3]. Household ownership of at least one LLIN also reached 69% in 2015 [3]. In Nigeria, most ITNs owned by households are LLINs, this is reflected in the matching proportions of households owning an ITN (68.8%) and households owning an LLIN (68.7%) in 2015 [3]. This indicates that the ITNs households own are LLINs. Therefore, in this study, both LLINs and nets that have been soaked with insecticides in the past 12 months will be referred to as ITNs.

To complement the ITN distribution efforts, the NMEP has also implemented advocacy, communication and social mobilization (ACSM) campaigns to improve knowledge of malaria prevention and control practices, create demand and increase the use of ITNs. Through these efforts, ITN use among the general population, children under 5, and pregnant women has increased significantly since 2008 [3, 7].

Despite the improvements observed in ITN ownership and use at the national level, large gaps remain to reach the goals of the NMSP 2014–2020 of universal access to ITNs and 80% ITN use among targeted populations by 2020 [8]. The increase in ITN ownership and use has also been uneven, with differences seen in household ITN ownership and use by place of residence, region of the country and household wealth quintile [3]. This study was conducted to guide future ITN strategies and investments to maximize impact, increase proportionate coverage and enhance progress toward the universal access target of all populations at risk of malaria. It examined sub-national profiles of household ownership of ITNs and ITN use in the general population to identify the best predictors of ITN ownership, use and which specific sub-groups to target.

## Methods

### Study design

This cross-sectional study consisted of a secondary analysis of the 2015 Nigeria Malaria Indicator Survey (NMIS). The outcomes of interest were household ownership of at least one ITN and ITN use in the general population. The 2015 NMIS was conducted between October and November 2015. The National Population Commission developed the sampling frame used for the survey, which represented all 36 states of Nigeria. For more information on the NMIS, see http://www.DHSprogram.com.

### Statistical analysis

The analysis was conducted in three phases.

*Phase 1: Selecting potential predictors* The authors calculated percentage estimates and conducted Chi square tests to assess the associations among each of the outcomes of interest and sociodemographic variables. Variables that were significantly associated with the outcomes of interest were retained as predictor variables. The individual and household-level variables were gender and age of the child, women’s age, women’s education level, parity, mother’s education level, education level of household head, place of residence, region, household wealth quintile, household size, and number of ITNs owned by the household.

*Phase 2: Running Chi square automatic interaction detector (CHAID) modelling* The authors ran a CHAID model for each outcome of interest. CHAID modelling uses a multi-level successive fitting algorithm. At each level, the model identifies the predictor variable that has the strongest association with the outcome of interest. The output of the analysis is a hierarchical tree with various levels (or branches), allowing for a visual representation of the relationship among the predictor variables and the outcome of interest. The first level of the tree represents the predictor variable that has the strongest interaction with the outcome of interest; this is also considered the root node. The root node is split into parent nodes (or leaves) for each category of the predictor variable that is significantly different based on a significance level of p < 0.05. Each parent node is further divided into child nodes by variables with a significant association until no further significant predictors are observed in the various subgroups. These end nodes are considered terminal nodes [9].

For each terminal node, the model provides information on the node percentage, the gain percentage, the response percentage, and the gain index percentage. The node percentage represents the demographic weight of the node in the sample. The gain percentage is the proportion of overall cases in the sample represented in the node. The response percentage refers to the proportion of cases with the outcome of interest in the node. The gain index percentage is the ratio of the response percentage in the node and of the overall sample. Gain index percentages greater than 100% represent a higher probability of experiencing the outcome of interest compared with the overall population [9].

For the CHAID models, the following criteria were used: a maximum tree depth of three levels, a minimum of 100 cases per parent node, and a minimum of 50 cases per child node. To identify the sub-groups that had lower coverage or uptake of interventions, the authors set the target category for the CHAID models to those who did not receive the intervention (e.g., household does not own an ITN) or did not practice the behaviour (e.g., ITN use).

*Phase 3: Running a logistic regression* The authors ran multiple logistic regression models for each outcome of interest with the most significant predictor variable identified in the CHAID analysis to estimate the odds ratio (OR). Select background characteristics that were assessed in the first phase of the analysis were controlled for in each model.

## Results

### Household ownership of ITNs

#### Household ownership of ITNs by sociodemographic characteristics

Household ownership of at least one ITN varied significantly by region, with the highest ownership observed in the North West region (90.6%; p < 0.0001) and the lowest ownership observed in the South West region (53.0%; p < 0.0001). ITN ownership was inversely proportional to household wealth quintile and education level of the household head, with the highest coverage observed in the lowest wealth quintile (86.1%; p < 0.0001) and among those with no formal education (72.0%; p < 0.001). Household ownership also varied significantly by place of residence and household size, with the highest ownership among households in rural areas (72.7%; p < 0.001) and large-size households (eight or more members) (83.2%; p < 0.0001) (Table 1).

**Table 1:** Percentage of household ownership of at least one ITN by background sociodemographic characteristics

Background characteristic	ITN ownership (%)	Chi square *p* value
Education level of the household head		0.0004
None	72.0	
Primary	71.8	
Secondary or higher	64.9	
Place of residence		0.0004
Urban	63.0	
Rural	72.7	
Region		< 0.0001
North Central	55.4	
North East	79.6	
North West	90.6	
South East	64.0	
South South	63.9	
South West	53.0	
Household wealth quintile		< 0.0001
Lowest	86.1	
Second	73.5	
Middle	68.7	
Fourth	64.2	
Highest	57.7	
Household size		< 0.0001
1–4 members	62.0	
5–7 members	72.4	
8 or more members	83.2	

#### Profiles of household ITN ownership

**Fig. 1:**
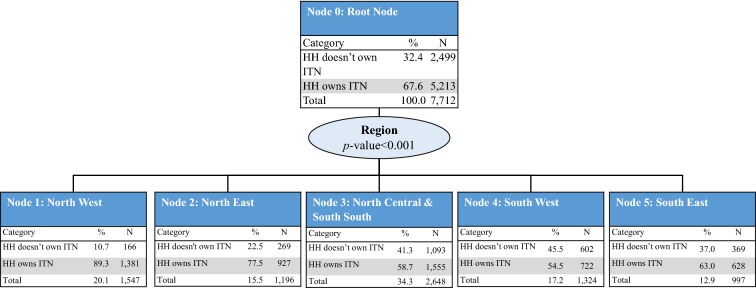
CHAID tree diagram for household ownership of at least one ITN.* HH* households,* N* number of households

To identify the sociodemographic profile of household ownership of at least one ITN, the five variables with a significant association with ITN ownership were included in the CHAID model: education level of the household head, region, place of residence, household wealth quintile, and household size. The model retained all five variables and consisted of 31 nodes, 19 of which were terminal nodes. Region of the country was the strongest predictor of household ITN ownership (p < 0.001) and was split into five parent nodes: North West (Node 1), North East (Node 2), North Central and South South (Node 3), South West (Node 4), and South East (Node 5). Household ITN ownership was observed to be the highest in the North West region (89.3%) and the lowest in the South West region (54.5%) (Fig. 1).

**Fig. 2:**
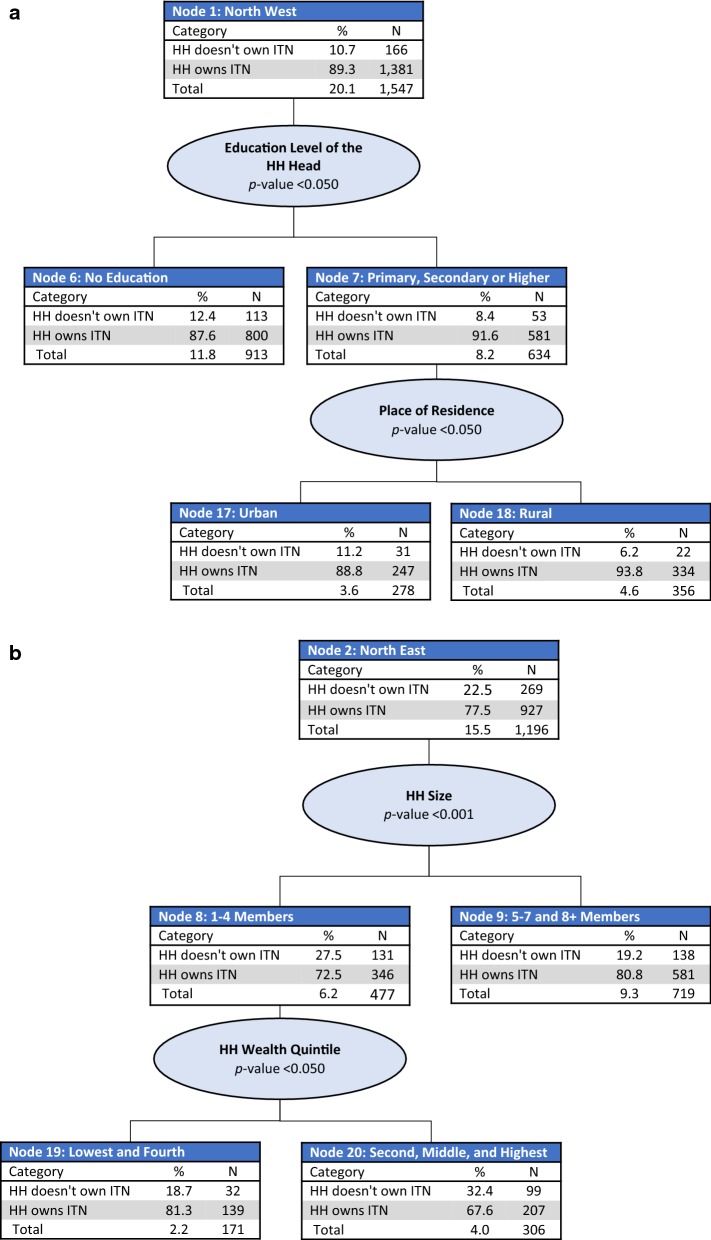

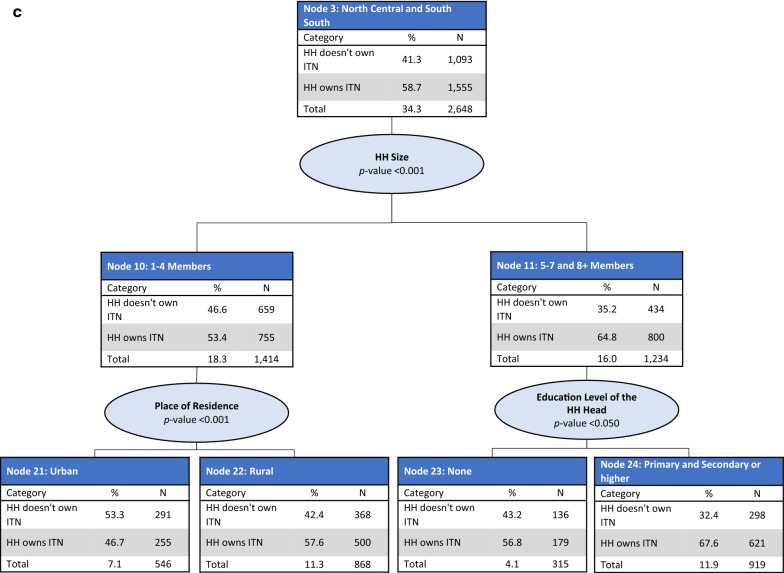

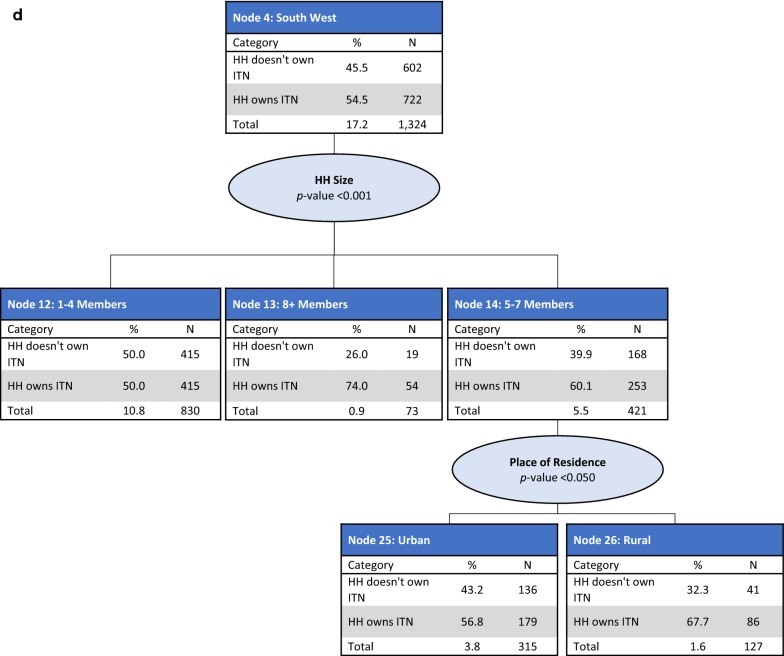

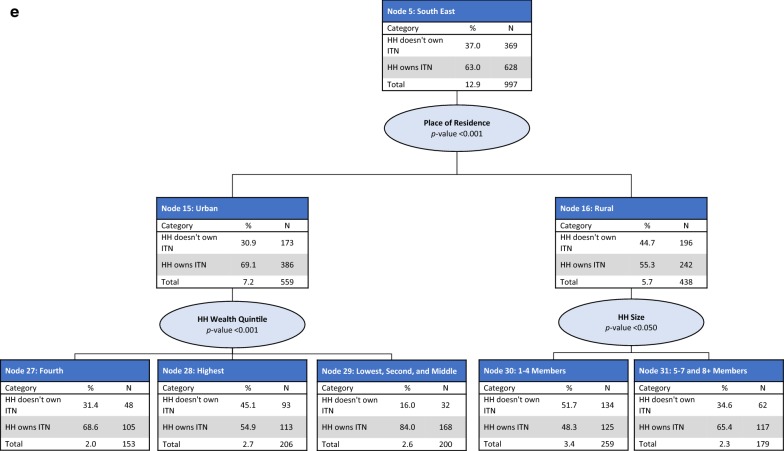
CHAID tree diagram for household ownership of at least one ITN. **a** North West,** b** North East,** c** North Central and South South,** d** South West regions and** e** South East region

*Node 1: North West region* For households in the North West region, education level of the household head was the strongest predictor of ITN ownership (p < 0.05) and was split between households in which household heads had no education (Node 6) and those in which household heads had a primary or higher level of education (Node 7). Households in which household heads had no education had slightly less ITN ownership (87.6%) compared with those in which household heads had some form of education (91.6%). Place of residence was the next best predictor among households in which household heads had some form of education (p < 0.05); among those, household ITN ownership remained high in both urban and rural areas, at 88.8% in urban areas (Node 17) and 93.8% in rural areas (Node 18) (Fig. 2a).

*Nodes 2–4: North East, North Central and South South, and South West regions* For households in the North East (Node 2), North Central and South South (Node 3) and South West (Node 4) regions, household size was the best predictor of ITN ownership (p < 0.001). In the North East, households were split into small-size households (one to four members) (Node 8), and medium (five to seven members) and large-size households (eight or more members) (Node 9). Lower ITN ownership coverage was observed in small-size households (72.5%) compared with medium and large-size households (80.8%). Among small-size households in the North East, wealth index was also a significant predictor of ITN ownership (p < 0.05) (Fig. 2b). Households in the North Central and South South regions were split by household size, with lower coverage observed in small-size households (Node 10, 53.4%) compared with those in medium to large-size households (Node 11, 64.8%). In small-size households, place of residence was a further significant predictor of ITN ownership (p < 0.001). In medium and large-size households, education level of the household head was a further significant predictor of ITN ownership (p < 0.05) (Fig. 2c). The South West region had three splits: small-size households (Node 12), medium-size households (Node 14), and large-size households (Node 13). The lowest ITN coverage was observed in small-size households (50.0%) and the highest in large-size households (74.0%). In medium-size households, place of residence was a further significant predictor of ITN ownership (p < 0.05) (Fig. 2d).

*Node 5: South East region* For households in the South East region, place of residence was the best predictor of ITN ownership (p < 0.001), with higher ownership observed in urban areas (Node 15, 69.1%) compared with rural areas (Node 16, 55.3%). In urban areas, household wealth quintile was a further significant predictor of ITN ownership (p < 0.001). ITN ownership was highest in households in the lowest three wealth quintiles (84.0%). In rural areas, household size was a further significant predictor of ITN ownership (p < 0.05), split into small-size households (Node 30, 48.3%) and medium to large-size households (Node 31, 65.4%) (Fig. 2e).

### Gain index

The gain index results for household ITN ownership highlight four sub-groups to target, based on their overall contribution to the proportion of households that do not own an ITN (gain percentage). These sub-groups are small-size households in the South West region (Node 12, gain: 16.6%), small-size households in the North Central and South South regions in both urban and rural areas (Node 21, gain 11.6%; Node 22, gain: 14.7%), and medium and large-size households in the North Central and South South regions with a household head education level of primary or higher (Node 24, gain: 11.9%). These four sub-groups represent 55% of all households that do not own ITNs out of the whole population, but they account for 41% of the overall population (Table 2).

**Table 2:** CHAID gain index for household ownership of at least one ITN

Node	Description of node	Node		Gain		Do not own ITN %	Index
		N	%	N	%		
21	North Central and South South regions; small-size households; urban area	546	7.1	291	11.6	53.3	164.5
30	South East region; rural area; small-size households	259	3.4	134	5.4	51.7	159.7
12	South West region; small-size households	830	10.8	415	16.6	50.0	154.3
28	South East region; urban area; highest wealth quintile	206	2.7	93	3.7	45.1	139.3
25	South West region; medium-size households; urban area	294	3.8	127	5.1	43.2	133.3
23	North Central and South South regions; medium and large-size households; no formal education	315	4.1	136	5.4	43.2	133.2
22	North Central and South South regions; small-size households; rural area	868	11.3	368	14.7	42.4	130.8
31	South East region; rural area; medium and large-size households	179	2.3	62	2.5	34.6	106.9
24	North Central and South South regions; medium and large-size households; primary or higher level of education	919	11.9	298	11.9	32.4	100.1
20	North East region; small-size households; highest, middle, and second wealth quintiles	306	4.0	99	4.0	32.4	99.8
26	South West region; medium-size households; rural area	127	1.6	41	1.6	32.3	99.6
27	South East region; urban area; fourth wealth quintile	153	2.0	48	1.9	31.4	96.8
13	South West region; large-size households	73	0.9	19	0.8	26.0	80.3
9	North East region; medium and large-size households	719	9.3	138	5.5	19.2	59.2
19	North East region; small-size households; lowest and fourth wealth quintiles	171	2.2	32	1.3	18.7	57.8
29	South East region; urban area; lowest three wealth quintiles	200	2.6	32	1.3	16.0	49.4
6	North West region; no formal education	913	11.8	113	4.5	12.4	38.2
17	North West region; primary or higher level of education; urban area	278	3.6	31	1.2	11.2	34.4
18	North West region; primary or higher level of education; rural area	356	4.6	22	0.9	6.2	19.1

### Multiple logistic regression for household ownership of an ITN and region

The odds of a household owning an ITN decreased significantly in the southern regions compared with the northern regions, with the greatest odds of owning an ITN in the North West (OR = 5.47, 95% CI 4.46–6.72, p < 0.001) and North East (OR = 2.32, 95% CI 1.94–2.77, p < 0.001) regions compared with the North Central region (Table 3). There was also a significant association between household ITN ownership and education level of the household head, household wealth quintile, and household size. Households in which the household head had a primary education (OR = 1.54, 95% CI 1.31–1.79, p < 0.001) or secondary or higher education (OR = 1.38, 95% CI 1.20–1.59, p < 0.001) had greater odds of owning an ITN compared with households in which the household head had no education. Medium-size households (OR = 1.49, 95% CI 1.33–1.67, p < 0.001) or large-size households (OR = 1.91, 95% CI 1.62–2.25, p < 0.001) had greater odds of owning an ITN compared with small-size households. In addition, all households in the lower wealth quintiles had greater odds of owning an ITN compared with households in the highest wealth quintile, with the greatest odds of owning an ITN among households in the lowest wealth quintile (OR = 2.08, 95% CI 1.59–2.72, p < 0.001) (Table 3).

**Table 3:** Multiple logistic regression of household ownership of at least one ITN

Explanatory variable	Odds ratio (95% CI)
Education level of the household head	
None	1.00 (reference)
Primary	1.54 (1.31–1.79)
Secondary or higher	1.38 (1.20–1.59)
Place of residence	
Urban	1.00 (reference)
Rural	0.97 (0.86–1.10)
Region	
North Central	1.00 (reference)
North East	2.32 (1.94–2.77)
North West	5.47 (4.46–6.72)
South East	1.35 (1.13–1.61)
South South	1.20 (1.02–1.41)
South West	1.03 (0.88–1.21)
Household wealth quintile	
Highest	1.00 (reference)
Fourth	1.25 (1.08–1.45)
Middle	1.55 (1.31–1.84)
Second	1.40 (1.13–1.73)
Lowest	2.08 (1.59–2.72)
Household size	
1–4 members	1.00 (reference)
5–7 members	1.49 (1.33–1.67)
8 or more members	1.91 (1.62–2.25)

### ITN use in the general population

#### ITN use by sociodemographic characteristics

ITN use in the general population varied significantly by region, with the highest use observed in the North West region (54.4%; p < 0.0001) and the lowest use in the South West region (21.1%; p < 0.0001) (Table 4). As with household ITN ownership, ITN use was inversely proportional to household wealth quintile and education level of the household head. The highest ITN use was observed among households in the lowest wealth quintile (52.7%; p < 0.0001) and whose household head had no formal education (40.8%; p < 0.0001). ITN use was also significantly higher among those living in rural areas (42.1%; p < 0.0001) and among large-size households (eight or more members) (41.7%; p < 0.001) (Table 4).

**Table 4:** Percentage of ITN use in the general population by background sociodemographic characteristics

Background characteristic	ITN use (%)	Chi square p-value
Education level of the household head		< 0.0001
None	40.8	
Primary	34.4	
Secondary or higher	27.6	
Place of residence		< 0.0001
Urban	29.3	
Rural	42.1	
Region		< 0.0001
North Central	30.2	
North East	45.4	
North West	54.4	
South East	21.2	
South South	28.9	
South West	21.1	
Household wealth quintile		< 0.0001
Lowest	52.7	
Second	44.7	
Middle	39.6	
Fourth	27.8	
Highest	21.9	
Household size		< 0.0001
1–4 members	35.6	
5–7 members	34.8	
8 or more members	41.7	

#### Profiles of ITN use in the general population

**Fig. 3:**
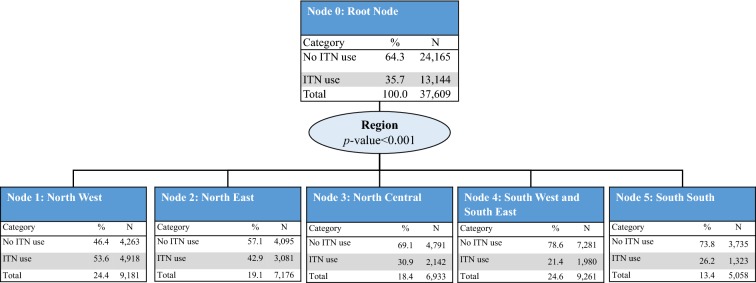
CHAID tree diagram for the general population who did not use an ITN the previous night.* HH* households,* N* number of households

The CHAID model included the following predictor variables: education level of the household head, region, place of residence, household wealth quintile, and household size. The final model retained all predictor variables and consisted of 55 nodes, 35 of which were terminal nodes. Region of the country was the best predictor of ITN use (p < 0.001) and was split into five parent nodes: North West (Node 1), North East (Node 2), North Central (Node 3), South West and South East (Node 4), and South South (Node 5). ITN use in the general population was the lowest in the South West and South East regions (21.4%) and the highest in the North West region (53.6%) (Fig. 3).

**Fig. 4:**
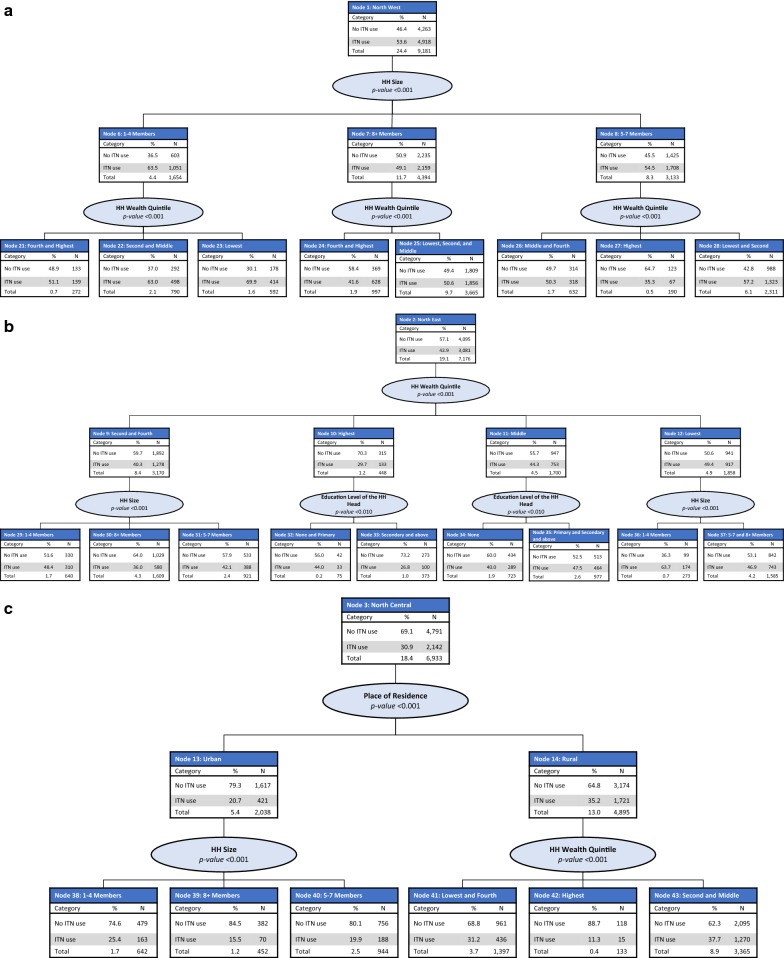

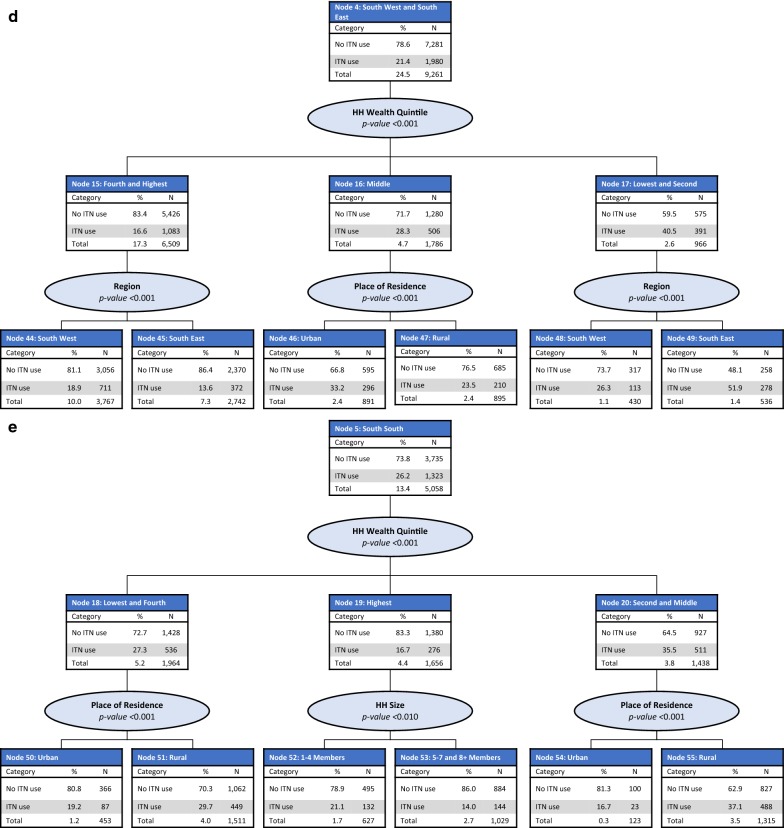
CHAID tree diagram for the general population who did not use an ITN the previous night:** a** North West,** b** North East,** c** North Central,** d** South West and South East and** e** South South region

*Node 1: North West region* In the North West region (Node 1), household size was the most significant predictor of ITN use (p < 0.001), split between small-size (Node 6), large-size (Node 7), and medium-size (Node 8) households. ITN use was highest among those living in small-size households (63.5%) and lowest among those living in large-size households (49.1%). In small, medium, and large-size households, household wealth quintile was the most significant predictor of ITN use (p < 0.001), with significantly higher ITN use observed among those living in households from the lower wealth quintiles (Fig. 4a).

*Node 2: North East region* In the North East region (Node 2), household wealth quintile was the most significant predictor of ITN use in the general population (p < 0.001), split among the second and fourth wealth quintiles (Node 9), the highest wealth quintile (Node 10), the middle wealth quintile (Node 11), and the lowest wealth quintile (Node 12). Among those living in households in the second and fourth wealth quintiles (Node 9) and the lowest wealth quintile (Node 12), a further significant predictor of ITN use was household size (p < 0.001 for both nodes). Among those from households in the middle and highest wealth quintiles (Nodes 10 and 11), a further significant predictor of ITN use was the head of household education level (p < 0.01 for both nodes) (Fig. 4b).

*Node 3: North Central region* In the North Central region (Node 3), place of residence was the most significant predictor of ITN use (p < 0.001), with significantly higher use observed among those living in rural areas (Node 14, 35.2%) compared with urban areas (Node 13, 20.7%). In urban areas, household size was a further significant predictor of ITN use (p < 0.001). Household wealth quintile was also a further significant predictor of ITN use among those living in rural areas (p < 0.001), with the highest ITN use observed among those from households in the second and third wealth quintiles (Node 43, 37.7%) and the lowest among the highest wealth quintile (Node 42, 11.3%) (Fig. 4c).

*Node 4: South West and South East regions* In the South West and South East regions (Node 4), household wealth quintile was the most significant predictor of ITN use (p < 0.001). Household wealth was split into three nodes: the top two wealth quintiles (Node 15), the middle wealth quintile (Node 16), and the lowest two wealth quintiles (Node 17). Significantly higher ITN use was observed among those from the lowest two wealth quintiles (40.5%). Among those from the top two wealth quintiles (Node 15) and those from the lowest two wealth quintiles (Node 17), region of the country was a further significant predictor of ITN use (p < 0.001), split between those from the South West and South East regions. Among those from the middle wealth quintile (Node 16), place of residence was a further significant predictor of ITN use (p < 0.001) (Fig. 4d).

*Node 5: South South region* In the South South region (Node 5), household wealth quintile was the most significant predictor of ITN use (p < 0.001), split between the lowest and fourth wealth quintiles (Node 18), the highest wealth quintile (Node 19), and the second and middle wealth quintiles (Node 20). The highest ITN use was observed among those from the second and middle wealth quintiles (Node 20, 35.5%). Among those from the lowest and fourth wealth quintiles and the second and middle wealth quintiles, place of residence was the most significant predictor of ITN use (p < 0.001 for both nodes). Among the highest wealth quintile, household size was the most significant predictor of ITN use (p < 0.01) (Fig. 4e).

### Gain index

The gain index for ITN use in the general population highlights two sub-groups with the largest proportion of non-ITN users: those from the South South region in the fourth and lowest wealth quintiles in the urban areas (Node 50, gain: 12.6%) and those from the South East region in the top two wealth quintiles (Node 45, gain: 9.8%). Other sub-groups with low ITN use in the general population are those from the North Central region in the second and middle wealth quintiles in rural areas (Node 43, gain: 8.7%); those from the South South region in the fourth and lowest wealth quintiles in rural areas (Node 51, gain: 4.4%); those from the North East region in the fourth and second wealth quintiles and from large-size households (Node 30, gain: 4.3%); and those from the North Central region in the fourth and lowest wealth quintiles in rural areas (Node 41, gain: 4.0%) (Table 5).

**Table 5:** CHAID gain index for ITN use in the general population

Node	Description of node	Node		Gain		Did not use ITN (%)	Index
		N	%	N	%		
42	North Central; rural; highest wealth quintile	133	0.4	118	0.5	88.7	138.1
45	South East region; top two wealth quintiles	2742	7.3	2370	9.8	86.4	134.5
53	South South region; highest wealth quintile; medium and large-size households	1029	2.7	885	3.7	86.0	133.9
39	North Central region; urban area; large-size households	452	1.2	382	1.6	84.5	131.5
44	South West region; top two wealth quintiles	123	0.3	100	0.4	81.3	126.5
50	South South region; fourth and lowest wealth quintiles; urban area	3767	10.0	3056	12.6	81.1	126.3
40	North Central region; urban area; medium-size households	453	1.2	366	1.5	80.8	125.7
54	South South region; middle and second wealth quintiles; urban area	944	2.5	756	3.1	80.1	124.6
52	South South region; highest wealth quintile; small-size households	627	1.7	495	2.0	78.9	122.9
47	South West and South East regions; middle wealth quintile; rural area	895	2.4	685	2.8	76.5	119.1
38	North Central region; urban area; small-size households	642	1.7	479	2.0	74.6	116.1
48	South West region; lowest two wealth quintiles	430	1.1	317	1.3	73.7	114.7
33	North East region; highest wealth quintile; secondary or higher education level	373	1.0	273	1.1	73.2	113.9
51	South South region; fourth and lowest wealth quintiles; rural area	1511	4.0	1062	4.4	70.3	109.4
41	North Central region; rural area; fourth and lowest wealth quintiles	1397	3.7	961	4.0	68.8	107.1
46	South West and South East regions; middle wealth quintile; urban area	891	2.4	595	2.5	66.8	103.9
27	North West region; medium-size households; highest wealth quintile	190	0.5	123	0.5	64.7	100.8
30	North East region; fourth and second wealth quintiles; large-size households	1609	4.3	1029	4.3	64.0	99.5
55	South South region; middle and second wealth quintiles; rural area	1315	3.5	827	3.4	62.9	97.9
43	North Central region; rural area; middle and second wealth quintiles	3365	8.9	2095	8.7	62.3	96.9
34	North East region; middle wealth quintile; no formal education	723	1.9	434	1.8	60.0	93.4
24	North West region; large-size households; lowest three wealth quintiles	729	1.9	426	1.8	58.4	90.9
31	North East region; fourth and second wealth quintiles; medium-size households	921	2.4	533	2.2	57.9	90.1
32	North East region; highest wealth quintile; none or primary level of education	75	0.2	42	0.2	56.0	87.2
37	North East region; lowest wealth quintile; medium and large-size households	1585	4.2	842	3.5	53.1	82.7
35	North East region; middle wealth quintile; no formal education	977	2.6	513	2.1	52.5	81.7
29	North West region; large-size households; lowest three wealth quintiles	640	1.7	330	1.4	51.6	80.2
26	North West region; medium-size households; fourth and middle wealth quintiles	632	1.7	314	1.3	49.7	77.3
25	North West region; large-size households; lowest three wealth quintiles	3665	9.7	1809	7.5	49.4	76.8
21	North West region; small-size households; top two wealth quintiles	272	0.7	133	0.6	48.9	76.1
49	South East region; lowest two wealth quintiles	536	1.4	258	1.1	48.1	74.9
28	North West region; medium-size households; lowest two wealth quintiles	2311	6.1	988	4.1	42.8	66.5
22	North West region; small-size households; middle and second wealth quintiles	790	2.1	292	1.2	37.0	57.5
36	North East region; lowest wealth quintile; small-size households	273	0.7	99	0.4	36.3	56.4
23	North West region; small-size households; lowest wealth quintile	592	1.6	178	0.7	30.1	46.8

#### Multiple logistic regression of ITN use in the general population

Compared with the North Central region, the general population in the North East (OR = 1.57, 95% CI 1.32–1.86, p < 0.001) and North West (OR = 2.04, 95% CI 1.73–2.41, p < 0.001) regions had greater odds of using an ITN. The general population in the South East (OR = 0.64, 95% CI 0.52–0.79, p < 0.001) and South West regions (OR = 0.75, 95% CI 0.62–0.90, p < 0.05) had lower odds of ITN use. Significant associations were also observed among ITN use in the general population and education level of the household head and household wealth quintile, with greater odds of ITN use among households in which the household head had a primary level of education (OR = 1.2, 95% CI 1.03–1.39, p < 0.05) compared with no education, and among those in the lower wealth quintiles compared with those in the highest wealth quintiles. The lowest wealth quintile had greater odds (OR = 3.26, 95% CI 2.53–4.20, p < 0.001) of ITN use compared with the highest wealth quintile (Table 6).

**Table 6:** Multiple logistic regression of ITN use by the general population

Explanatory variable	OR (95% CI)
Education level of the household head	
None	1.00 (reference)
Primary	1.20 (1.03–1.39)
Secondary or higher	1.13 (0.99–1.30)
Place of residence	
Urban	1.00 (reference)
Rural	0.98 (0.85–1.11)
Region	
North Central	1.00 (reference)
North East	1.57 (1.32–1.86)
North West	2.04 (1.73–2.41)
South East	0.64 (0.52–0.79)
South South	1.02 (0.85–1.22)
South West	0.75 (0.62–0.90)
Household wealth quintile	
Highest	1.00 (reference)
Fourth	1.57 (1.32–1.87)
Middle	2.59 (2.13–3.14)
Second	2.61 (2.08–3.27)
Lowest	3.26 (2.53–4.20)
Household size	
1–4 members	1.00 (reference)
5–7 members	0.93 (0.83–1.05)
8 or more members	0.98 (0.85–1.14)

## Discussion

This study examined sub-national profiles of household ownership of at least one ITN and ITN use in the general population. The CHAID analysis indicates that region of the country is the best predictor of both household ITN ownership and ITN use. Other significant predictors of both outcomes are household size, head of household education level, household wealth quintile, and place of residence.

The heterogenous transmission of malaria across Nigeria may best explain why region of the country is the most significant predictor. The NMEP focuses its ITN distribution efforts in areas with a high malaria burden. Across the three northern regions, the prevalence of malaria in children under five ranges from 26 to 37% compared with 14 to 19% in the three southern regions [3, 10, 11]. The NMEP uses free mass distribution of ITNs in these areas, a strategy that has been proven to dramatically increase household ITN ownership [12–14]. Possession of ITNs through these means has been shown to increase ITN use [15]. The NMEP’s ITN distribution strategy is coupled with ACSM activities, which previous studies found to significantly generate demand for and increase the use of ITNs [16–18]. As to the regional differences in household ITN ownership, the concentration of ACSM activities may contribute to the disproportionate use of ITNs. Household ITN ownership and use were observed to be the highest in the northern regions compared with the southern regions, with differences across regions. The regional differences are also supported by the multiple logistic regression findings, with the odds of a household owning an ITN being five times greater in the North West region compared with the North Central region.

The ITN distribution strategy may also account for the disproportionate ITN ownership across household sizes. Medium to large-size households were found to have higher ownership in the North East, North Central, South South, and South West regions compared with small-size households. de Beyl et al. noted a similar result, which found that larger size households were more likely to register and receive ITNs [19]. In addition to mass distribution campaigns, the NMEP uses other distribution channels, such as schools, ANC and immunization clinics. Employing these channels has been shown to help maintain ITN coverage and reduce inequities among vulnerable populations [20, 21]. However, use of these channels leaves a gap in coverage for households without young children or a woman who has attended ANC in the past 2 years to obtain an ITN [22].

Despite higher ITN ownership among medium to large-size households, ITN use was greater in small-size households compared with large-size households. These results were comparable with recent studies, which found very low intra-household saturation with ITNs among large households following mass distribution campaigns [13, 23–26]. Higher use among smaller households may be indicative of larger households not having enough ITNs to cover everyone in the household. Several studies have discovered that improving household access and having sufficient ITNs were significant predictors of ITN use [14, 24–28]. Households with higher levels of education generally had higher ITN ownership, although this was a significant predictor in the North Central, South South and North West regions only; comparable gaps were found in other studies examining equity in coverage [29, 30].

The NMEP has increased its focus on equity among households in the lowest wealth quintiles and in rural areas. Children in the lowest wealth quintiles had a disproportionately greater burden of malaria (64%) compared with children in the highest wealth quintiles (13%) [3]. For children in rural areas, the prevalence of malaria was significantly higher (56%) compared with children in urban areas (24%) [3]. There is an overlap between these populations, and among those living in rural settings, more than 60% are in the lowest two wealth quintiles. Among those living in urban settings, more than 75% are in the top two wealth quintiles [4]. Analogous to other countries with a high malaria burden, to help increase ITN ownership across these households, the NMEP employed free ITN distribution [12, 15, 31–33]. This has led to households in both the lowest wealth quintiles and in rural areas having higher ITN ownership [3]. Most households in rural areas were more likely to own an ITN, except for those in the South East region, where households in urban areas had higher ITN ownership. There was variability in ITN use across urban and rural settings. In the South West and South East regions, ITN use was higher in urban areas, and the opposite was observed in the North Central and South South regions. This variance may be linked to the higher ITN ownership found in the urban areas of the South East region and in the rural areas of the North Central and South South regions. Higher ITN use among those in the lowest wealth quintiles and in rural areas reflects the NMEP’s achievements in reducing inequity, because other research has found these populations to have the lowest ITN ownership and use [12, 34].

The CHAID analysis aids in the understanding of variables that affect household ITN ownership and ITN use in the general population. Attention to the sub-groups identified in this research study may help Nigeria progress toward achieving the goal of universal ITN coverage and increase the overall use of ITNs in the general population. The gain index table provides specific information on the sub-groups to better inform intervention strategies. Efforts focused on increasing net ownership in small-size households in the South West (Node 21), North Central (Node 12), and South Central (Node 22) regions should be considered, as these areas include 43% of households not owning an ITN, though accounting for 29% of the population. To increase ITN use in the general population, increased focus needs to be placed on sub-groups containing households in the lowest and fourth wealth quintiles in the urban areas of the South South region (Node 45), the top two wealth quintiles in the South East region (Node 50), and the second and middle wealth quintiles in rural areas of the North Central region (Node 43), which account for 31% of the population that do not use ITNs.

### Limitations

This study was a secondary data analysis of the 2015 NMIS and was limited to the data available. The NMIS sampling methodology was not designed for multiple stratifications, resulting in smaller sample sizes for this analysis, which may have limited the number of nodes and the identification of other potential significant predictors. Future studies may assess further potential predictors of ITN ownership and use beyond the sociodemographic characteristics that were examined in this study.

## Conclusions

Nigeria has made significant progress in expanding ITN coverage and generating demand for and use of ITNs over the past decade. Despite these efforts, large gaps remain to achieve universal ITN access and 80% ITN use. This study reveals the regional differences in ITN ownership and use throughout Nigeria and identifies specific subgroups among small-size households in the South West, North Central and South Central regions. It also identified specific sub-groups among households in the lowest and wealthiest quintiles in the southern regions that could be targeted and fill the coverage and use gaps.

## Data Availability

The data that support the findings of this study are available from the Demographic and Health Surveys (DHS) Programme upon reasonable request and with permission of the DHS Programme. The datasets analysed during the current study are available in the DHS repository, https://dhsprogram.com/data/available-datasets.cfm.

## Abbreviations

ACSM: advocacy, communication, and social mobilization
ANC: antenatal care
CHAID: Chi square automatic interaction detector
CI: confidence interval
NMEP: National Malaria Elimination Programme
NMIS: Nigeria Malaria Indicator Survey
NMSP: National Malaria Strategic Plan
OR: odds ratio
IRB: institutional review board approval
ITN: insecticide-treated net
USAID: United States Agency for International Development
WHO: World Health Organization
